# Brain-Derived Neurotrophic Factor (BDNF) as a Predictor of Treatment Response in Schizophrenia and Bipolar Disorder: A Systematic Review

**DOI:** 10.3390/ijms252011204

**Published:** 2024-10-18

**Authors:** Andrés Liberona, Natalia Jones, Karen Zúñiga, Verónica Garrido, Mario Ignacio Zelada, Hernán Silva, Rodrigo R. Nieto

**Affiliations:** 1 Escuela de Medicina, Facultad de Medicina, Universidad de Chile, Santiago 8380453, Chile; 2 Clínica Psiquiátrica Universitaria, Hospital Clínico de la Universidad de Chile, Universidad de Chile, Santiago 8380453, Chile; 3 Departamento de Psiquiatría y Salud Mental Norte, Facultad de Medicina, Universidad de Chile, Santiago 8380453, Chile; 4 Departamento de Neurociencias, Facultad de Medicina, Universidad de Chile, Santiago 8380453, Chile

**Keywords:** BDNF, neurotrophins, biomarkers, schizophrenia, bipolar disorder, treatment refractoriness, treatment response, treatment resistance

## Abstract

Brain-derived neurotrophic factor (BDNF) is a potential biomarker of response to treatment in psychiatric disorders. As it plays a role in the pathophysiological development of schizophrenia and bipolar disorder, it is of interest to study its role in predicting therapeutic responses in both conditions. We carried out a systematic review of the literature, looking for differences in baseline BDNF levels and the Val66Met BDNF polymorphism in these disorders between responders and non-responders, and found information showing that the Val/Val genotype and higher baseline BDNF levels may be present in patients that respond successfully to pharmacological and non-pharmacological treatments. However, there is still limited evidence to support the role of the Val66Met polymorphism and baseline BDNF levels as predictors of treatment response.

## 1. Introduction

Precision medicine aims to offer the most appropriate therapeutic options according to the profile of each patient, based on the measurement and characterization of objectifiable variables [1]. In psychiatry, the diagnostic and therapeutic process requires subjective evaluation and interpretation by a treating physician of symptoms referred by patients [2]. Furthermore, current classification systems allow for clinical and biological heterogeneity within diagnostic constructs. This has implications for the correct understanding of treatment response results in clinical trials. Given the above, it has been of special interest in psychiatry to determine objectifiable variables, including biological markers, that could identify subgroups of patients—within a diagnosis—and predict responses to different treatments, in order to move towards a more personalized form of medicine within this specialty.

Research on biomarkers could lead to a better way of classifying, diagnosing, or predicting the course of psychiatric diseases [3]. One biomarker frequently postulated in the literature is Brain-Derived Neurotrophic Factor (BDNF), a member of the neurotrophins family that plays a fundamental role in the neurogenesis, growth, differentiation, survival, and plasticity of neuronal networks, with a consequent impact on cognitive functions, such as learning and memory [4]. It has been demonstrated that decreased levels of BDNF can be associated with a series of pathologies of the neurodegenerative sphere, as well as neuronal death [5]. At the same time, due to its cerebral origin, it could play an important role in psychopathology, as well as its treatment. A recent systematic review carried out by our group summarized the roles of circulating levels of BDNF and BDNF-related polymorphisms (notably Val66Met) as biomarkers of treatment response in in patients with Major Depressive Disorder (MDD) [6]. Similarly, there is abundant research describing the relationship of BDNF with other psychiatric disorders, mostly in terms of pathophysiology, but also as a biomarker predictor of response to treatment, notably Schizophrenia (SCZ) and Bipolar Disorder (BD).

SCZ is a chronic psychiatric disorder characterized by positive (hallucinations and delusions), negative (flattened affectivity), and cognitive (memory or attention deficits) symptoms [7]. Since alterations in neurodevelopment are important in the pathogenesis of SCZ, BDNF, as a neurotrophin relevant for neurodevelopment, is an interesting biomarker candidate in this illness [8]. Originally, an association was described between altered levels of BDNF and its receptor at the brain level in patients with SCZ, specifically in mesolimbic areas [9]. It has been described that BDNF plays an important pathophysiological role in SCZ, impacting the survival and plasticity of dopaminergic, cholinergic, and serotonergic neurons [10]. Since then, the relationship between BDNF and SCZ has been extensively studied, and several studies have measured its levels in clinical populations, with dissimilar results [11]. Thus, a meta-analysis found that serum BDNF levels are reduced in patients with SCZ, although heterogeneity was found in results across different studies [11]. On the other hand, there are controversial findings regarding the BDNF levels in patients receiving antipsychotic treatment, as some studies have found no differences between groups [12,13], others have found a lower level in those who are clozapine users [14,15], and others have found a higher level after several months of atypical antipsychotic treatment [16]. Whether BDNF is a marker of response to available treatments for SCZ is a less explored topic.

BD is a chronic mood disorder characterized by the recurrence of manic/hypomanic and depressive episodes [17]. BDNF has been shown to be an important contributor to the neuroplastic changes described in patients with this disorder, demonstrating that serum levels decrease in depressive and manic episodes, returning to normal levels in euthymia [18]. However, BDNF levels tend to decrease as BD progresses over the course of several years and multiple episodes [18]. Lithium is a mood stabilizer par excellence, which has made it a cornerstone in the treatment of BD. A review published in 2021 that sought to address the roles of various genes in terms of response to lithium treatment distinguished the BDNF gene as a relevant agent; however, this highlights the conflicting results of the literature addressed, recommending that its conclusions be evaluated with caution [19]. Given the above, it has been a focus of interest to correlate BDNF with its therapeutic response, among other pharmacological and non-pharmacological approaches.

It is worth noting that both disorders share common genetic and neuropathological findings, so recent research lines have encouraged their joint study, being able to understand SCZ, BD, and schizoaffective disorder as projections of a spectrum of interrelated psychiatric disorders [20]. This has been expressed in the development of several approaches that seek to reclassify these disorders based on the biotypes of patients according to their biological substrates, instead of only clinical characteristics, an integrative view that could allow for better identifying their dysfunctions, as well as providing better diagnostic and therapeutic tools [21].

In this context, the present review seeks to identify and analyze the available evidence on the predictive role of BDNF and its polymorphisms in terms of response to treatments for SCZ and BD.

## 2. Materials and Methods

This review was conducted between November 2022 and March 2024. It did not require submission to or approval by an ethics committee, since it corresponds to a systematic review of the literature published in this field of research. In order to guarantee the quality of the reviewed articles, they were selected exclusively from indexed databases.

The systematic search was conducted in the PubMed database, selecting original research articles, meta-analyses, and narrative and systematic reviews published in English or Spanish up to March 2024. To ensure the inclusion of all relevant studies, a broad search strategy was employed, using the concepts “BDNF” AND “Predicts” AND “Treatment” AND “Response”. From this search, which encompassed all medical conditions in which BDNF was evaluated, only those with results related to SCZ/psychosis, BD, and schizoaffective disorder were selected. This initial broad strategy enabled us to identify all possible studies evaluating these specific disorders, including those categorized more broadly under terms like “Mental Health” or “Psychiatric Disorders”, without explicitly mentioning them in the title or abstract. Table 1 shows the combination of keywords used in this database.

Due to the identification of studies not included in the initial search among the references of the articles selected, three additional independent systematic searches were conducted in the PubMed database for each psychiatric condition (SCZ/psychosis, BD, and schizoaffective disorder). Original research articles, meta-analyses, and narrative and systematic reviews published in English or Spanish up to March 2024 were selected. The concepts chosen for this second search were “BDNF” AND “Treatment” AND “Resistance”, along with the following specific keywords for each condition: (1) AND “Schizophrenia” OR “Psychosis”, (2) AND “Bipolar Disorder”, (3) AND “Schizoaffective Disorder”. This narrower, more specific strategy allowed us to identify relevant studies that used the term “Resistance” instead of “Response”. The keyword “Predicts” was initially included, but was ultimately excluded from the MeSH terms, because successive comparative searches showed that it narrowed the results, potentially omitting relevant studies. Only after a meticulous comparison of the retrieved results for each condition and confirming that excluding “Predicts” added more studies was the decision made to leave it out. The combination of keywords used in this second search are also shown in Table 1.

The inclusion criteria were as follows: Empirical or primary studies, with reviews focused on the relationship between BDNF and response/resistance to treatments of SCZ, BD, and schizoaffective disorder. The target population was human beings with no defined age limit and included quantitative, qualitative, or mixed studies published in Spanish or English (Table 2).

**Figure 1:**
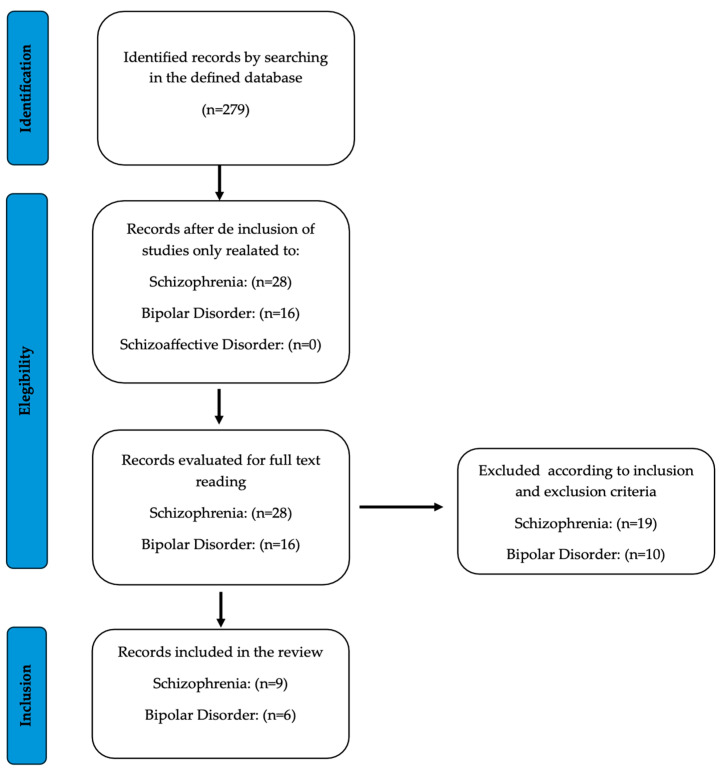
Flow chart of the literature search and study selection process in a systematic review of the literature on the relationship between BDNF and response to treatment of SCZ, BD, and schizoaffective disorder. Articles published up to March 2024.

**Figure 2:**
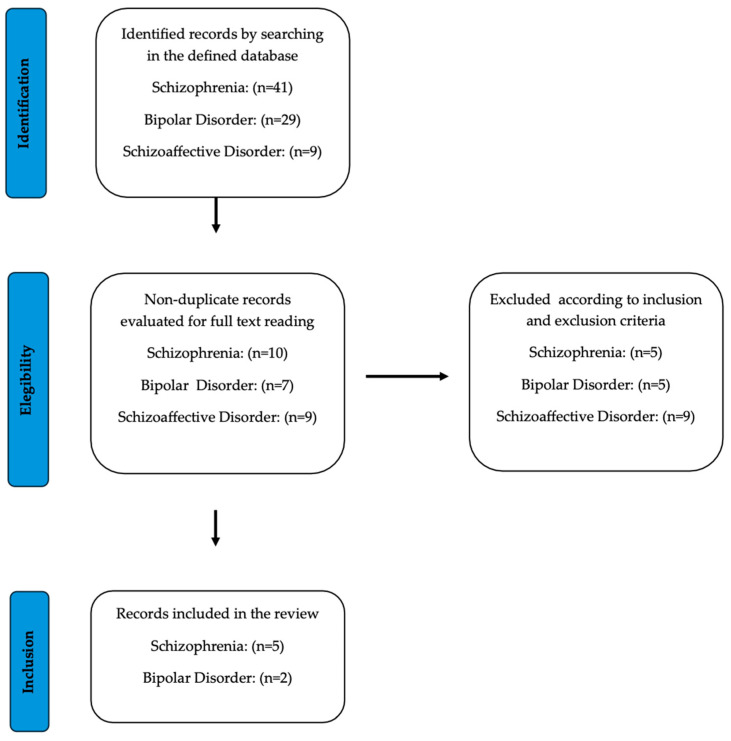
Flow chart of the literature search and study selection process in a systematic review of the literature on the relationship between BDNF and resistance to SCZ, BD, and schizoaffective disorder treatment. Articles published up to March 2024.

The PRISMA guidelines were followed to collect and filter the information. Initially, a total of 279 articles were obtained from the specified database. After excluding articles based on inclusion criterion No. 1, 28 studies were selected for abstract screening for SCZ and 16 articles were selected for BD. No articles on schizoaffective disorder met the proposed criteria. The total number of studies was equally and randomly distributed among five reviewers, who were instructed to read the full articles, determine the final inclusions, and synthesize the results and main conclusions in a Google Drive spreadsheet. Doubts and disagreements were resolved through discussion, and the final selection was made by consensus among the reviewers and an additional sixth reviewer who only had access to the spreadsheet (Figure 1). Regarding the second search, 41 articles were initially retrieved for SCZ/psychosis, 29 for BD, and 9 for schizoaffective disorder. These articles were then screened by title and abstract, and only non-duplicate records were selected for full-text review (Figure 2). In sum, a total of 14 (Table 3) and 8 (Table 4) articles were finally incorporated for SCZ and BD, respectively.

## 3. Results

### 3.1. Schizophrenia

#### 3.1.1. BDNF Levels and Response to Pharmacological Treatments in Schizophrenia

In patients with SCZ that underwent 6 weeks of treatment with risperidone, the baseline BDNF levels were significantly lower in non-response patients than others. After treatment, much-improved patients had significantly higher plasma BDNF than non-response patients [24]. In relation to treatment with clozapine, one study evaluated patients who had been taking this antipsychotic for at least 18 months and were users of a stable daily dose for at least 4 weeks prior to measurements, showing a higher serum BDNF level among patients classified as responders to antipsychotic treatment with clozapine compared to non-responders to treatment [31].

A recent study, published in 2023, sought to determine the role of BDNF as a predictor of response in terms of the improvement of psychotic symptoms in patients with SCZ [34]. In total, 89 patients with a first episode of SCZ were recruited, together with 90 controls, distinguishing between early and late responders, defining 2 weeks of treatment as the cut-off, and applying the Positive and Negative Symptoms Scale (PANSS) to quantify improvements [34]. During that time, they received treatment with risperidone, showing a decrease in the PANSS score directly proportional to the BDNF levels in the early response group, which was not evidenced in late responders, in turn, evidencing the role of this biomarker as an independent predictor of response to treatment after performing a regression analysis [34].

#### 3.1.2. BDNF Levels and Response to Non-Pharmacological Treatments in Schizophrenia

Regarding electro-convulsive therapy (ECT), it has been reported that baseline BDNF levels, as well as increments in BDNF levels after this intervention, could be considered as predictors of a good clinical outcome, operationalized as a reduction in PANSS questionnaire scores [32]. However, in a different publication, no significant correlations between baseline BDNF levels and treatment responses were found in a combined group of patients who underwent both ECT and antipsychotics treatments [30]. A recent meta-analysis published in the year 2023, which incorporated six studies in this regard, evidenced conflicting results regarding the variability of BDNF after ECT treatment in patients with treatment-resistant SCZ, showing a significant increase in only two of the studies analyzed [35]. On the other hand, the other four studies also showed an increase in the biomarker, although this was not statistically significant [35]. The authors inferred that this could be explained in the context of cohorts that incorporate treatment-resistant patients, who could present a more latent increase in the biomarker compared to responders, as well as confounding factors such as the size of the studies and the simultaneous use of other antipsychotics [35].

#### 3.1.3. BDNF Polymorphisms and Treatment Response in Schizophrenia

It has been reported in the literature that the BDNF genetic variant Val66Met (rs6265) is associated with a differential response to antipsychotic treatment, as carriers of Val/Val genotype show a better response to clozapine [22,26] and olanzapine [28]. However, other studies have found no association between the presence of these polymorphisms and clinical improvement [25], in addition to presenting no association between the BDNF G196A and C270T polymorphisms with response to treatment with neuroleptics or the risk of SCZ [23]. However, these findings could be due to alterations in other polymorphisms of the BDNF functioning system; for example, it was reported that no association was found with BDNF polymorphisms and response to treatment with clozapine, but there was an association with the polymorphisms rs1778929 and rs10465180 of the NTRK2 gene [29], which corresponds to the high-affinity receptor for BDNF. Another study analyzed the prevalence of the Val66Met polymorphism in clozapine users, considering them as a treatment-resistant group due to the failure of two previous antipsychotics before initiating clozapine therapy [27]. This study found that the presence of homozygous and heterozygous Val66Met variants was more frequent in the clozapine group than in the non-clozapine group [27]. The association was stronger in homozygous carriers and appeared to be dose-dependent [27].

Regarding responses to other forms of non-pharmacological treatment, a study was conducted using neuronavigation-guided repetitive transcranial magnetic stimulation (rTMS) treatment in veteran patients with SCZ. A total of 4 weeks of stimulation was performed in the dorsolateral prefrontal cortex of one group and another received placebo stimulation. The rTMS treatment generated an improvement in immediate memory performance compared to the control group. Importantly, there was a greater improvement in memory in those patients with homozygous CC for the rs12273539 polymorphism in the BDNF gene compared to those carrying the T allele (TT or CT), who did not show a significant improvement compared to their baseline performance [33].

### 3.2. Bipolar Disorder

#### 3.2.1. BDNF Levels and Response to Pharmacological Treatments in Bipolar Disorder

Two different review studies identified correlations between lithium responders and serum levels of BDNF, describing that excellent lithium responders (patients in whom monotherapy prevented future episodes of BD for 10 or more years) maintained normal serum levels, even in the long term [41,42].

Similarly, there has been research conducted on prophylactic lithium treatment in patients with BD. In 2010, a study was conducted with 141 euthymic patients with BD treated with long-standing prophylactic lithium therapy, divided into the following three groups: excellent lithium responders, partial lithium responders, and lithium non-responders. The three groups of patients were compared with a control group of healthy subjects. The lithium non-responders were found to have significantly lower levels compared to the control group subjects [38]. In this same study, no association was found between the age of the patients or the duration of BD with BDNF levels. This study contrasts with a previous study, conducted in 2006, which also studied prophylactic lithium therapy and concluded that the factors investigated (including BDNF) were not predictive for a prophylactic effect [37].

#### 3.2.2. BDNF Levels and Response to Non-Pharmacological Treatments in Bipolar Disorder

Among the non-pharmacological treatments described for BD, a prospective longitudinal study conducted in 2020 stands out, whose purpose was to identify the predictive roles of different variables in the effectiveness of psychoeducation in BD, including BDNF among the elements to be evaluated. Due to research limitations, this biomarker was only measured in 54 of 90 participants; however, when performing a univariate analysis, it was identified that those individuals with higher baseline BDNF levels presented a tendency to respond positively to psychoeducation, which was attributed to the neuroprotective effect of the biomarker. A post-treatment increase among responders was also observed [43].

#### 3.2.3. BDNF Polymorphisms and Treatment Response in Bipolar Disorder

A studied carried out in 2011 found that the presence of the Val allele was associated with a lower probability of being an excellent responder to lithium prophylaxis [39]. A 2013 review concluded that there was no significant correlation between the Val66Met polymorphism and lithium response in patients with BD, despite an odds ratio of 2.67 (*p* = 0.078) when comparing perfect responders versus non-responders [40], but it did find significant differences when comparing excellent responders and partial responders against non-responders [40]. Another study, which retrospectively analyzed the response to lithium carbonate therapy in patients with BD, found no significant differences in the genotypic distribution or allele frequency of the Val66Met polymorphism between responders and non-responders to therapy [36].

## 4. Discussion

Psychiatric disorders are characterized for affecting the behavioral, mental, and affective states of people in a wide variety of ways, causing significant personal, social, and economic impacts in people who suffer from them, as well as an important burden for health systems and societies, which is aggravated by their high prevalence and incidence, as well as their delayed diagnosis and associated stigmas, among other factors. In addition to the above, this group of disorders is extremely complex due to the immense number of genetic, neurobiological, psychological, relational, and social factors involved in their presentation and pathophysiology. This means that it is not yet clear how these various factors interact with each other to produce the florid symptomatologic range that characterizes these disorders. For the same reason, there is a general lack of knowledge about the mechanisms of functioning of the treatments used, both pharmacological and non-pharmacological, as well as a clear lack of markers to predict the level of response that particular individuals or groups of patients will have to standard medical therapy.

The aim of this study was to answer the question of whether BDNF, either through its peripheral blood levels or polymorphisms, has a predictive capacity regarding the effectiveness of different treatment strategies in SCZ, BD, and schizoaffective disorder, similar to the MDD study conducted by our group, who recently published an article addressing the same question [6]. The evidence is controversial regarding the usefulness of BDNF as a marker of response and seems to depend largely on the pathological area in question.

Regarding BDNF levels and SCZ, in terms of pharmacological management, all the reviewed studies analyzing plasma levels [24,31,34] found that lower BDNF levels predicted a poorer response, apparently due to changes in BDNF release, as well as a disruption in the dendritic targeting of BDNF mRNA [44]. However, the three studies varied significantly in the specific thresholds that could be used. This highlights a lack of sufficient evidence to establish any definitive conclusions about whether serum BDNF levels can be used to predict treatment response in clinical settings. Further research is needed to replicate these findings and evaluate different doses, treatment schedules, durations, and specific drugs [24]. When considering non-pharmacological schemes such as ECT, at least four studies identified increases in serum BDNF levels after treatment (either combined or in monotherapy), of which three found a positive correlation with symptomatic improvement (or negative with symptom persistence) [45] and the remaining one found that this increase in BDNF could not explain the symptomatologic improvement, since pharmacological treatment alone also decreased the severity of symptoms without altering serum BDNF levels [46]. In this line of research, there seems to be a greater relationship between BDNF and response to treatment, however, studies analyze post-treatment and not pre-treatment levels, which makes it difficult to generate a predictive value for BDNF levels regarding response. On the other hand, there is no time cut-off criterion in the studies analyzed that would allow for early classifications of those who will respond in the long term and those who will not, although this could be easier to analyze with the data obtained in the studies. Other studies have evaluated the safety of ECT use in patients with SCZ refractory to treatment, using as a marker of safety the increase in BDNF levels that occurs more in combined therapy (with drugs and ECT) than with monotherapy [45]. Another group found that only those patients treated with ECT saw increased BDNF levels, but not those treated with drug therapy, although both saw a decrease in the severity of their symptoms, concluding that changes in BDNF levels are not related to clinical improvement [46]. Despite this, one study analyzed the predictive value of BDNF levels in patients receiving combined ECT and pharmacological therapy. This study did not find any significant difference in the baseline plasma BDNF levels between responders and non-responders; however, the BDNF levels were lower in both groups before treatment [30].

With respect to genetic variations, at least three studies support the conception that the Val66Met (rs6265) polymorphism may predict a worse response to antipsychotic treatment in patients with SCZ [22,26,28], although this was not a completely consistent finding across studies, given that other research groups have reported finding a non-association [25]. It is important to remember that, while most studies focus their analysis on rs6265 (also called G196A), several other SNPs on the BDNF gene have been less consistently studied and are, thus, not yet sufficient for drawing solid conclusions. As for adverse effects, the Val66Met variant was associated (potentially due to serotonergic effects) with weight gain at 6 years of treatment but not at 6 and 11 weeks [45]. In turn, this polymorphism is associated with lower basal glucose levels [45]. It appears that this variant has some relationship with IL-1B that should be further explored [46]. Further studies are needed to elucidate the roles these polymorphisms, as well as other genetic alterations, may have in predicting treatment response.

The usefulness of BDNF as a biomarker of response in other domains such as physical and cardiovascular health has also been evaluated. In a 12-week exercise program for patients with SCZ, it was observed that the increase in serum BDNF levels post-intervention correlated positively with cardiovascular fitness and leg strength, although other aspects of improvement in relation to the symptoms of the pathology were not evaluated [47].

In relation to the measurement of BDNF levels as a marker of response in the treatment of BD, studies were mainly focused on the use of lithium. There is a relevant correlation between maintaining normal BDNF levels and an excellent response to lithium treatment, both therapeutically [33,41,48] and prophylactically [42], while the presence of the Val66Met polymorphism in some studies was associated with a greater probability of being a good responder [43], which contrasts with the findings regarding SCZ, in which the presence of this polymorphism was associated (inconsistently) with a worse response to pharmacological treatment [22,26,28]. However, other investigations have concluded that the presence of the Val66Met polymorphism and serum BDNF levels do not correlate with the response to lithium [39], which again shows the lack of replicability and consistency in studies on the association between BDNF polymorphisms and response to treatment.

In relation to other treatments for BD, the use of ketamine generated a significant reduction in BDNF levels [49], which would head in the opposite direction with respect to the other results, since, in general, an increase in BDNF, and not its reduction, is associated with symptomatic improvements. For example, in the study that used psychoeducation as a non-pharmacological treatment, it was shown that individuals with higher baseline BDNF levels presented a tendency to respond positively to therapy, which, in turn, was related to a post-treatment increase among responders [37]. This again reinforces the idea that higher BDNF levels are related to a better clinical state. Regarding other treatments less used in BD, such as recombinant erythropoietin (EPO), one study explored the effect of EPO on peripheral BDNF levels in patients with affective disorders, and found that it decreased plasma BDNF levels in patients with treatment-resistant depression, but no effect on these levels was observed in patients with BD treated with EPO [50].

Studies that have only evaluated BDNF levels have found lower levels in patients with SCZ in comparison with healthy controls and patients with BD [51], while others have found reduced values to a similar extent in both groups with respect to controls [52,53]. With respect to polymorphisms, there are studies that have not found an association between the presence of the Val66Met polymorphism and the presentation of either of the two disorders [54], while other studies have found variations in at least other two polymorphisms (rs10835210 CA and rs11030101 AT) in both disorders [25] in comparison to control subjects. On the other hand, a study that evaluated the presence of different mRNA types in the post mortem brains of patients with SCZ, BD, and MDD found no significant differences in the total transcripts among the three groups, but did find specific regional differences in mRNA subtypes according to each group, suggesting a complex modulation between the levels of gene interaction and regulatory mechanisms that could be specific to each pathology and brain region [55]. Therefore, further studies and a joint analysis of both pathologies are still required to establish a clearer link regarding the role of BDNF and its different forms in both disorders. In this sense, studies could be carried out in relation to the role of BDNF antisense molecule polymorphisms that regulate and control BDNF expression, which have shown relevant associations with SCZ and BD studied together [56].

With respect to schizoaffective disorder, it has been reported in the literature that there is a lower serum BDNF level with respect to control subjects in a similar proportion to patients with SCZ [57]. When considering polymorphisms, different studies have related the presence of the Val66Met polymorphism more frequently in patients with this disorder with respect to controls and even more frequently than in patients with SCZ [58]. However, in spite of being a relevant psychiatric diagnosis and closely related to those previously described, no evidence was found in the search for the study of BDNF as a marker of response to treatment that separates this clinical group from patients with SCZ, since, in many of the studies found, although they spoke of both disorders, their sample was composed mainly of the first type of patient and their conclusions made few allusions to schizoaffective disorder [59,60]. This opens up this path as an opportunity for future research.

It stands out from the literature review that there is still much dissidence between the results of different studies, even within the same psychiatric pathology. This is largely due to the fact that, despite an apparent abundance of studies on the topic, there are actually few investigations that have similar methodologies, groups, and objectives that make them easy to compare. Most studies evaluate slightly different aspects of the variety of ways of analyzing the topic. Thus, for example, some authors evaluate the relation between BDNF levels and responses to different pharmacological treatments [12,16], use different peripheral blood samples (serum and plasma), or focus on pre-treatment or post-treatment levels [34], which makes it relatively difficult to compare between studies due to the excessive combinatorial possibilities. The main difficulty generated by the above is that it amplifies the methodological or sampling weakness of certain studies in that, since there are few replicable studies under similar conditions, it is difficult to discern whether each study presents findings that effectively correspond to the underlying biology of the disorders.

On the other hand, the same scientific production regarding psychiatric pathologies does not escape from the fundamental problems of psychiatric diagnostic classification based on phenomenological and clinical descriptions of symptomatologic groupings [61], rather than on clearly identified pathophysiological pathways. This implies the possible existence of different subtypes or biotypes within psychiatric disorders with different modes of expression and roles of BDNF within their etiopathological mechanisms. As an example of this, BDNF levels have been found to be different between SZ patients with better cognitive functioning in comparison to those with worse cognitive functioning [62,63].

It is important to remember the special structural and functional heterogeneity of the central nervous system and the role it plays, especially in mental health disorders. Thus, it is possible that there is a differential regulation of genes according to brain region, subpopulations, and even between different cell types. A study on peripheral samples, however, especially blood samples, generates a homogenization of the levels of different regions, subpopulations, or cell types [64], which could veil an important role of BDNF that is specific to one of the mentioned levels.

Among the limitations of this study is the lack of an analysis of the quality of the reviewed studies, treating them all as equivalent. Similarly, we did not incorporate mathematical analysis, which would have strengthened our conclusions beyond subjective interpretations of the study syntheses. We believe that a more focused study, concentrating on a single pathology and one BDNF source (either genetic SNPs or plasma/serum levels), could shed more light on this topic and provide more quantitative conclusions, potentially leading to actionable thresholds for BDNF values in clinical settings. Additionally, one of the methodological limitations is the absence of PROSPERO registration for this review. PROSPERO is an important international registration system for systematic reviews, helping researchers to avoid duplication [65]. However, we believe that, despite this being a significant issue, the lack of registration did not affect the quality of the search or the conclusions we reached.

Despite the limitations mentioned above, the use of biomarkers such as BDNF may open an important window for both differential diagnosis and early diagnosis, even in the pre-symptomatic stages of different disorders. However, it is unlikely that this objective can be achieved using a single molecule as a biomarker. In this sense, the use of different macromolecules together could achieve a greater accuracy either diagnostically or as a predictor of response [66]. In addition to their clinical utility, it is expected that the use of biomarkers such as BDNF will be able to provide interesting insight in terms of disease classification, clarifying the existence of different biotypes within each pathology, such as the efforts of the Research Domain Criteria (RDoC) [67], thus commencing a new era in the classification, diagnosis, prediction, and treatment of psychiatric pathologies.

On the other hand, much research has been performed on genotyping to identify the allelic variants that explain the prognostic variability in different disorders. This offers important diagnostic opportunities with a minimally invasive study for personalized medicine. However, as in the case of its protein form with a single marker, it will be difficult to estimate risk based on a single altered gene, especially when several patients present these alleles without presenting psychiatric pathology [68]. Genes such as BDNF may interact with other genes to define clinical features [69]. In this sense, scores based on polygenic risk that incorporate, through GWAS studies, several SNPs, that contribute all together for a clinical result, may once again play an important role in the future. Specific polygenic risk scores might be developed for treatment-resistant patients [70,71], where local studies representing patients from each part of the world will be necessary [72]. Alternatively, more dynamic visions coming from bioinformatics that incorporate gene co-expression networks or protein–protein interaction networks may shed light on how BDNF can play important roles in different clinical scenarios [73]. This could be helpful for improving the understanding of its relationships with other proteins and genes, and, therefore, enhancing the diagnostic and predictive performance of BDNF levels and polymorphisms.

## 5. Conclusions

The research regarding BDNF and treatment responses in SCZ and BD shows important heterogeneity across the different studies analyzed in this review. However, the search for biomarkers that complement the classical clinical descriptions and mental examinations is an actively growing field with important projections and interesting possibilities. There are still important limitations that will hopefully be overcome as technology and the number of studies increase, generating convergent evidence that will allow for meta-analyses and systematic reviews that will gradually yield definitive conclusions, and from there, contribute to guidelines and recommendations with clinical utility that will support the work of professionals, ultimately generating benefits for future patients.

## Figures and Tables

**Table 1:** Identification and expansion of the search base concepts: algorithm applied in the database.

First Search	Second Search
“BDNF”	“BDNF”
“Predicts”	“Treatment”
“Treatment”	“Resistance”
“Response”	“Schizophrenia” OR “Psychosis”, AND “Bipolar Disorder”, AND “Schizoaffective Disorder”

**Table 2:** Inclusion and exclusion criteria applied in the literature search.

Inclusion Criteria	Exclusion Criteria
Studies and reviews focused on the relationship between BDNF and response/resistance to treatment of SCZ, BD, and schizoaffective disorder.	Non-empirical or secondary studies, such as editorial publications, commentaries, and books.
Quantitative and mixed-methods studies with adequate definitions, reliable methods, operationalization of concepts, and data analysis.	Studies reporting research in non-human populations.
Studies available in the PubMed database.	Studies focused on the validation and/or construction of instruments.
Studies published until March 2024.	Studies that did not directly evaluate the predictive value of BDNF on outcomes.
Full text available in Spanish or English.	Studies that did not have a direct therapeutic intervention.

**Table 3:** Summary of the main results for the most commonly analyzed variables related to BDNF and Schizophrenia. Studies that found a significant difference in treatment response are signaled with + in effect (last column); those that did not find a significant difference in treatment response are signaled with n.s. A meta-analisis was included here, marked with an asterisk (*).

Author	Ref	Treatment	Sample	BDNF Non Responders	BDNF Responders	Effect
Hong CJ., et al., 2003	[22]	Pharmacologial (Clozapine)	Val66Met (rs6265)	Val/Met + Met/Met 80.6% (n = 29) Val/Val 19.4% (n = 7)	Val/Met + Met/Met 73.7% (n = 42) Val/Val 26.3% (n = 15)	+
Anttila S., et al., 2005	[23]	Pharmacological (Typical antipsychotics)	Val66Met (rs6265)	Val/Met + Met/Met 31.4% (n = 16) Val/Val 68.6% (n = 35)	Val/Met + Met/Met 30.2% (n = 13) Val/Val 69.8% (n = 30)	n.s.
Lee BH & Kim YK. 2009	[24]	Pharmacological (Risperidone)	Plasma	631.26 ± 300.81 pg/mL (n = 8)	1079.74 ± 484.60 pg/mL (n = 13)	+
Pae Cu., et al., 2012	[25]	Pharmacological	Val66Met	-	-	n.s.
Zai GC., et al., 2012	[26]	Pharmacologial (Clozapine)	Val66Met (rs6265)	Val/Met + Met/Met 44.7% (n = 34) Val/Val 55.2% (n = 42)	Val/Met + Met/Met 25.5% (n = 38) Val/Val 74.5% (n = 111)	+
Zhang JP., et al., 2013	[27]	Pharmacological (Not-Clozapine)	Val66Met (rs6265)	Val/Met + Met/Met 48.8% (n = 43) Val/Val 51.1% (n = 45)	Val/Met + Met/Met 29.3% (n = 56) Val/Val 70.7% (n = 135)	+
Nikolac PM., et al., 2014	[28]	Pharmacologial (Olanzapine)	Val66Met (rs6265)	Val/Met + Met/Met 46.7% (n = 42) Val/Val 53.4% (n = 48)	Val/Met + Met/Met 32.2% (n = 40) Val/Val 67.7% (n = 84)	+
Mitjans M., et al., 2015	[29]	Pharmacologial (Clozapine)	Val66Met (rs6265)	-	-	n.s.
Li J., et al., 2016	[30]	Pharmacological and Non-pharmacological (ECT)	Plasma	9.500 ± 2.600 pg/mL (n = 74)	9.800 ± 2600 pg/mL (n = 116)	n.s.
Krivoy A., et al., 2018	[31]	Pharmacological (Clozapine)	Plasma	1668 ± 820 pg/mL (n = 35)	2066 ± 814 pg/mL (n = 54)	+
Li J., et al., 2020	[32]	Non-pharmacological (ECT)	Plasma	-	-	+
Su X., et al., 2023	[33]	Non-pharmacological (rTMS)	BDNF rs12273539	TT or CT	CC	+
Zhao T., et al., 2023	[34]	Pharmacological (Risperidone)	Plasma	1095.51 ± 264.45 pg/mL (n = 48)	1188.50 ± 182.50 pg/mL (n = 41)	+
Szota AM., et al., 2023 *	[35]	Non-pharmacological (ECT)	Plasma	-	-	n.s.

**Table 4:** Summary of the main results for the most commonly analyzed variables related to BDNF and Bipolar Disorder. Studies that found a significant difference in treatment response are signaled with + in effect (last column); those that did not find a significant difference in treatment response are signaled with n.s. A meta-analisis was included here, marked with an asterisk (*).

Author	Ref	Treatment	Sample	BDNF Non Responders	BDNF Responders	Effect
Masui T., et al., 2006	[36]	Pharmacological (Lithium)	Val66Met	Val/Val 31.3% (n = 16), Val/Met 52.9% (n = 27), Met/Met 15.6% (n = 8)	Val/Val 37.2% (n = 41), Val/Met 50% (n = 55), Met/Met 12.7% (n = 14)	n.s.
Michelon L., et al., 2006	[37]	Pharmacological (Lithium)	BDNF	TT 63.2% (n = 35) CT 34.7% (n = 17) CC 2.0% (n = 1)	TT 66.1% (n = 39) CT 32.2% (n = 19) CC 1.7% (n = 1)	n.s.
Suwalska A., et al., 2010	[38]	Pharmacological (Lithium)	Plasma	21.9 ± 15.5 ng/mL (n = 50)	26.7 ± 16.8 ng/mL (n = 30)	+
Rybakowski JK., et al., 2011	[39]	Pharmacological (Lithium)	Val66Met	Met/Val + Met/Met 24% Val/Val 76%	Met/Val + Met/Met 56% Val/Val 44%	+
Ehret MJ., et al., 2013 *	[40]	Pharmacological (Lithium)	Val66Met	Val/Met	Val/Val	+
Rybakowski JK. 2014a	[41]	Pharmacological (Lithium)	Val66Met	Val/Val	Val/Met	+
Rybakowski JK. 2014b	[42]	Pharmacological (Lithium)	Val66Met	Val/Val	Val/Met	+
Reinares M., et al., 2020	[43]	Non-pharmacological (Psycho-education)	Plasma	21.14 ± 10.35 ng/mL (n = 43)	27.37 ± 10.70 ng/mL (n = 47)	+
